# Cataract-causing αAG98R-crystallin mutant dissociates into monomers having chaperone activity

**Published:** 2011-01-05

**Authors:** Murugesan Raju, Puttur Santhoshkumar, K. Krishna Sharma

**Affiliations:** 1 Department of Ophthalmology, University of Missouri, Columbia, MO; 2 Department of Biochemistry, University of Missouri, Columbia, MO

## Abstract

**Purpose:**

The G98R mutation in αA-crystallin is associated with autosomal dominant cataract in humans. We have reported that mutant G98R protein has substrate-dependent chaperone activity. Further studies on this G98R mutant protein revealed that mutant protein shows reduced oligomeric stability and accelerated subunit dissociation at a low protein concentration. The purpose of present study was to investigate the chaperone function of dissociated subunits of αAG98R-crystallin.

**Methods:**

Substitution of glycine with arginine at position 98 in human αA-crystallin was accomplished by site-directed mutagenesis. The recombinant protein was expressed in *E .coli* cells and purified by chromatographic techniques. Purified αAG98R-crystallin was diluted to a concentration of 0.1 mg/ml in 50 mM phosphate buffer containing 150 mM NaCl (pH 7.2) and incubated at 37 °C for 24 h. The monomeric subunits were isolated from the oligomers through 50 kDa cutoff filters. The monomers were analyzed by SDS–PAGE, mass spectrometry, and circular dichroism spectroscopy and characterized by multi-angle light-scattering methods. Chaperone activity was tested against four client proteins: citrate synthesis, alcohol dehydrogenate, bovine βB2-crystallin and ovotransferrin.

**Results:**

Gel filtration studies showed that αAG98R-crystallin oligomers dissociate readily into monomers. Subunits of αAG98R-crystallin, isolated either by size exclusion chromatography or filtration showed chaperone activity against heat-denatured alcohol dehydrogenase, citrate synthase, bovine βB2-crystallin, and chemically denatured ovatransferrin. SDS–PAGE analysis of the mutant protein incubated at 37 °C for 12 days showed autolysis, which was confirmed by matrix-assisted laser desorption ionization time-of-flight mass spectrometry (MALDI TOF MS/MS) analysis of αAG98R-crystallin fragments recovered after SDS–PAGE.

**Conclusions:**

The present study shows that the G98R mutation in αA-crystallin produces unstable oligomers that dissociate into active chaperone subunits. The chaperone activity of the dissociated subunits against four client proteins suggests that the αA-crystallin subunits are the minimal units of chaperone activity.

## Introduction

α-Crystallin belongs to the family of small heat shock proteins (sHSP) and is composed of two subunits, αA- and αB-, which form heteromers and homomers with varying number of subunits [1]. Size exclusion chromatography (SEC) analysis of a wide range of αA- and αB-crystallin oligomer concentrations, from as low as 5 μg/ml to as high as 5 mg/ml, has shown that such oligomers, although dynamic, do not dissociate into monomers under physiologic conditions [2]. However, wild-type αA- and αB-crystallins have been found to dissociate into dimers and tetramers at <5 μg/ml concentrations [3]. A detailed study on a truncated form of αB-crystallin, Q151X, has shown that this mutant dissociates into monomers at <0.1 mg/ml concentrations, whereas it aggregates at higher concentrations [4].

Like other sHSPs, α-crystallin prevents aggregation of denaturing proteins [5]. It is well documented that both αA- and αB-crystallin subunits show chaperone activity [5]. Many of the mutant αA- and αB-crystallins studied thus far have shown altered structure, aggregation propensity and varying chaperone activity. Several mutations in the α-crystallin gene have been linked to cataract [6]. For example, an autosomal dominant G98R mutation in αA-crystallin, found in an Indian family, leads to pre-senile cataract in young adults [7]. Earlier studies with recombinant αAG98R-crystallin revealed that the mutation affects the protein’s structure, stability and chaperone activity [8]. In a previous study we showed that αAG98R-crystallin exhibits substrate-dependent chaperone activity [9].

αA- and αB-crystallin oligomers as well as the dimers have been found to exhibit chaperone activity [10]. The evidence in support of this comes from studies where native oligomeric crystallins or truncated human αB-crystallin (αB 57–157), which exists as dimer, were used in chaperone assays [11]. Those data suggest that oligomer organization is not essential for the chaperone property of α-crystallin subunits. We have shown that short sequences of αA- and αB-crystallin, representing the chaperone sites of native proteins, possess chaperone activity [12,13]. Chaperone activity has also been found in truncated αB-crystallin capable of dissociating into monomers [4]. While we were characterizing αAG98R-crystallin protein, we observed that the αAG98R-crystallin oligomer readily dissociates into monomeric form. This gave us an opportunity to investigate whether chaperone function is an inherent property of full-length individual subunits of αA-crystallin. In the present study, chaperone function of dissociated αAG98R-crystallin subunits was investigated using citrate synthesis (CS), alcohol dehydrogenate (ADH), bovine βB2-crystallin, and ovotransferrin as client proteins. The results suggest that the dissociated subunits exhibit chaperone activity in a concentration-dependant manner.

Recent studies have shown that the majority of mutant crystallins form insoluble aggregates, nonspecifically interact with other proteins and precipitate, yet some mutants and truncated forms of αA- and αB-crystallins are less stable and form oligomers with increased polydispersity [4,14-18]. Additionally, one of the mutant α-crystallins, αBR120G-crystallin, was shown to undergo premature or accelerated degradation on storage [19]. In the present study we show that although the mutant αAG98R-crystallin aggregates at high concentrations, at low concentrations the protein dissociates into monomers and undergoes autolysis on storage.

## Methods

### Preparation of αAG98R-crystallin mutant and wild-type αA-crystallin

Human αA-crystallin cDNA (obtained from J.M. Petrash, Washington University, St. Louis, MO) was cloned into pET-23d (+) vector (Novagen, Madison, WI). This cloned cDNA was used as a template to generate mutation in the αA-crystallin gene using a Quick Change Site Directed Mutagenesis kit (Stratagene, La Jolla, CA) with a set of primers as described earlier [9]. The G98R mutation was confirmed by automated DNA sequencing. Both mutant and wild-type proteins were expressed in *E.coli* BL21(DE3) pLysS cells (Invitrogen, Carlsbad, CA) and purified as described earlier [8]. Briefly, bacterial cell pellets were obtained from 1 l culture, suspended in 10 ml lysis buffer containing 50mM Tris-HCl (pH 8.0), 100 mM NaCl, and 2 mM EDTA, and treated with 50 μl of protease inhibitor cocktail III, 1 mg lysozyme, and 10 mM DTT. The cell suspension was treated with 1 μl of benzonase and incubated at 37 °C on a shaking platform for 30 min. The extract was centrifuged at 17,000× g for 1 h. The αAG98R-crystallin protein partitioned into insoluble fraction was washed and redissolved in 20mM Tris-HCl buffer (pH 7.2) containing 6 M urea and 1 mM EDTA. The urea-dissolved supernatant was filtered and loaded into a Q-Sepharose Fast Flow ion-exchange column equilibrated with Tris-EDTA buffer. The protein was eluted using a stepwise gradient of 1 M NaCl in 50 mM Tris-HCl (pH 7.2) containing 1 mM EDTA at a flow rate of 1 ml/min. The wild-type αA-crystallin protein partitioned into the soluble fraction was initially purified on a HiLoad 16/60 Superdex 200 gel filtration column equilibrated with 50 mM phosphate buffer containing 150 mM NaCl (pH 7.2). The peak containing the wild-type αA-crystallin was pooled, concentrated, treated with solid urea (6 M) and purified using anion exchange column, as described for the mutant protein. The purity of the proteins was examined by SDS–PAGE and the molecular mass was determined by mass spectrometry. The concentrations of the mutant and of the wild-type proteins were estimated using Bio-Rad protein assay reagent.

### Light scattering studies

Protein samples were injected into a TSKG5000PW_XL_ (Tosoh Bioscience, Montgomeryville, PA) size-exclusion column equilibrated with 50 mM phosphate buffer containing 150 mM NaCl (pH 7.2). The flow rate was 0.75 ml/min. The size-exclusion column was attached to a HPLC system connected with UV and refractive index detectors (Shimadzu, Columbia, MD) and coupled to static multi-angle laser light scattering (DAWN-EOS) and dynamic quasi-elastic light scattering detectors (Wyatt Technology, Santa Barbara, CA). The data were analyzed using ASTRA (5.3.4.14) software (Wyatt Technology).

### Isolation of αAG98R-crystallin monomer protein

Purified αAG98R-crystallin mutant protein was diluted in 50 mM phosphate buffer containing 150 mM NaCl (pH 7.2) to obtain a concentration of 0.1 mg/ml. This solution was incubated at 37 °C for 24 h and passed through 50 kDa cutoff membrane filters (Amicon, Millipore). Filtrate used in all experiments was analyzed by size exclusion chromatography, as described above.

### Mass spectrometric analysis

Dissociated subunits of αAG98R-crystallin mutant proteins were desalted using PepClean C18 column before mass spectrometry (MS). The protein bound to the PepClean column was eluted in 40 μl of 70% acetonitrile, dried on a SpeedVac, re-dissolved in 20 μl of elution buffer, and subjected to MALDI TOF MS analysis.

### Chaperone activity measurements

The chaperone activity of wild-type αA-crystallin and of the αAG98R-crystallin monomer protein was measured using substrates alcohol dehydrogenase (ADH; Biozyme, San Diego, CA), citrate synthese (CS; Sigma, St Louis, MO), βB2-crystallin, and ovotransferrin (Sigma, St. Louis, MO). The extent of aggregation was estimated by monitoring the light scattering at 360 nm using a Shimadzu UV-VIS spectrophotometer equipped with a temperature-controlled multi-cell transporter. Assays were done in the absence or presence of wild-type or mutant proteins as a function of time. Aggregation of ADH (75 μg) was induced by the addition of 100 mM EDTA in 50 mM phosphate buffer containing 150 mM NaCl (pH 7.3) at 37 °C. For CS aggregation assay, 75 μg of CS in 1 ml of 40 mM HEPES-KOH buffer (pH 7.4) was heated to 43 °C. CS aggregation at 360 nm was measured up to 1 h. During ovotransferrin aggregation assay, 100 μg of the protein in 1 ml of 50 mM phosphate buffer containing 150 mM NaCl (pH 7.2) was kept at 37 °C and allowed to denature. To investigate the effect of αAG98R-crystallin on βB2-crystallin, 150 μg of βB2-crystallin isolated from bovine lens extract was used. The aggregation assay was performed at 37 °C, as described earlier [20].

### Mutant protein stability studies

αAG98R-crystallin mutant protein (0.1 mg/ml) was incubated in 50 mM phosphate buffer containing 150 mM NaCl (pH 7.2) under sterile conditions for 0, 2, 6, and 12 days at 37 °C. Aliquots of these samples were subjected to SDS–PAGE and MS analysis. In addition, 250 μl of the sample was analyzed by size exclusion chromatography connected with MALS instrument. The data were analyzed as described above. Control experiments with wild-type αA-crystallin were run simultaneously with the mutant protein experiments.

## Results

### Expression of wild-type αA- and αAG98R-crystallins and isolation and characterization of αAG98R-crystallin subunits

**Figure 1:**
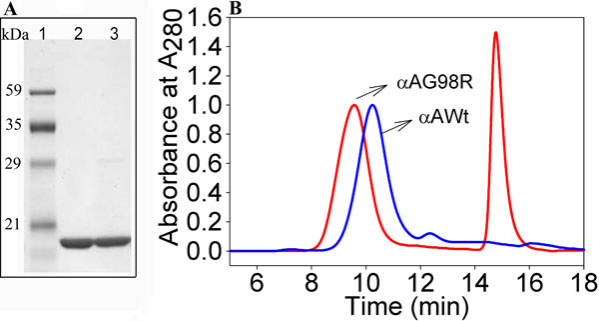
Electrophoretic and size exclusion chromatography profile of wild-type and αAG98R-crystallin. **A**: Comassie Blue–stained SDS–PAGE of G98R mutant and wild-type αA-crystallin, showing >98% purity of the crystallins used in the study. Lane 1, protein markers; Lane 2, wild-type αA-crystallin; and Lane 3 αAG98R-crystallin. **B**: Size exclusion chromatography profile of αAG98R-crystallin and wild-type αA-crystallin. 100 μg of protein at 1mg/ml concentration in phosphate buffer was injected into a TSK-G5000PW_XL_ gel filtration column (7.6 mm×30 cm). Fractions of 0.75 ml were collected. The mutant G98R protein (red) shows two peaks, one at 9.5 min, corresponding to the oligomer, and another peak at 15 min, corresponding to monomer mass. Wild-type α-crystallin (blue) did not show two peaks.

Recombinant proteins were expressed and isolated following the procedure described earlier [9]. Although the mutant protein expressed in *E.coli* cells forms inclusion bodies due to high in vivo protein concentration after urea solubilisation, purification and refolding at <2 mg/ml concentrations the recombinant protein remains soluble in assay buffers for several days to permit the characterization. On the basis of SDS–PAGE profile, both wild-type and mutant recombinant crystallins used in this study were >98% pure (Figure 1A). Size exclusion chromatography of mutant G98R protein at 1 mg/ml concentration gave an elution profile with an oligomer peak eluting at 9.5 min and a monomer peak eluting at 15 min, indicating the dissociation of oligomeric assembly (Figure 1B). In contrast, wild-type αA-crystallin eluted as a single peak at 10 min, corresponding to the elution time of αA-crystallin oligomers (Figure 1B).

**Figure 2:**
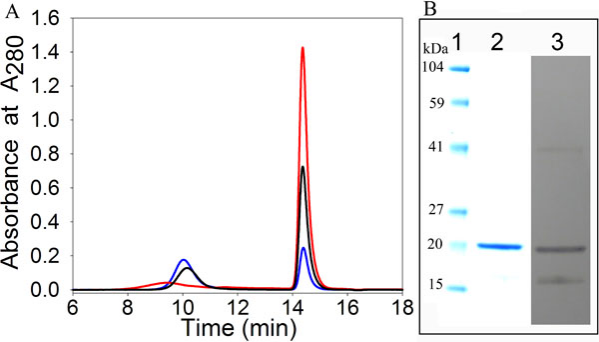
Size exclusion chromatography profile and immunoblot showing monomers of αAG98R-crystallin. **A**: Size-exclusion chromatography of three concentrations of αAG98R-crystallin. αAG98R-crystallin was injected to a TSK-5000 PW_XL_ column (7.6 mm×30 cm) in 3 concentrations and the elution profile was recorded by following the 280 nm absorbance. Blue, 0.5 mg/ml; Black, 0.2 mg/ml and Red, 0.1 mg/ml. **B**: SDS–PAGE and immunoblot of dissociated subunits of αAG98R-crystallin protein obtained during size exclusion chromatography analysis. Lane1, Marker proteins; lane 2, αAG98R-crystallin stained with Coomassie Blue; lane 3, western blot of lane 2 sample probed with anti-αA-crystallin.

**Figure 3:**
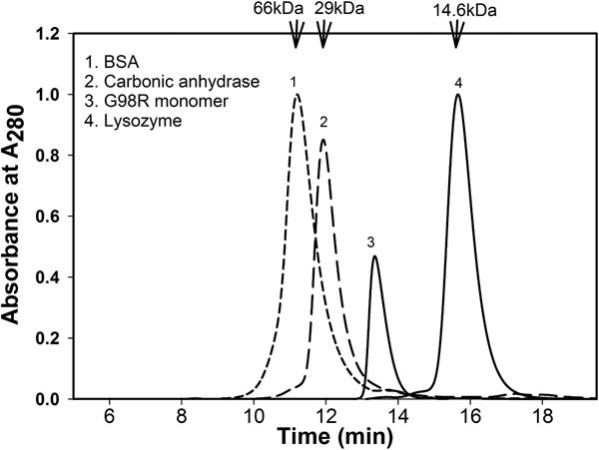
Relative elution profile of αAG98R-crystallin subunits during size exclusion chromatography on TSK-3000 PW_XL_ column (7.6 mm×30 cm). 1) BSA (66 kDa); 2) Carbonic anhydrase (29 kDa); 3) αAG98R-crystallin and 4) lysozyme (14.6 kDa).

After observing the dissociation of mutant protein during size exclusion chromatography, the following experiment was performed to isolate a sufficient amount of monomer protein for further study. Purified αAG98R-crystallin protein was diluted to concentrations of 0.1 mg/ml, 0.2 mg/ml and 0.5 mg/ml in 50 mM phosphate buffer containing 150 mM NaCl (pH 7.2). The diluted protein solutions were subjected to size exclusion chromatography. The elution profile showed an inverse relationship between the concentration of mutant protein and the degree of dissociation. Over 95% dissociation was observed when the protein concentration was 0.1 mg/ml (Figure 2A), whereas only partial dissociation occurred with higher αAG98R concentrations. Examination of the effect of pH on dissociation of αAG98R-crystallin mutant protein, at 1 mg/ml concentration, revealed about 70% dissociation of the mutant protein at pH 5.9. The precipitation of the mutant protein at higher concentrations did not allow us to fully evaluate the relationship between αAG98R-crystallin concentration and dissociation at pH 5.9 (data not shown). The dissociated subunits of αAG98R-crystallin were also analyzed by SDS–PAGE and western blot. The results showed that, in addition to a 20 kDa band corresponding to the molecular weight of αA-crystallin subunit, an additional band at ~11 kDa region was observed in immunoblots (Figure 2B). A corresponding protein band was not clearly visible, however, after staining with Coomassie Blue. When the immunoblot was analyzed using ImageJ software, the 11 kDa band was about 13% of the total. To investigate whether the 11 kDa protein band is a breakdown fragment of mutant protein or a contaminant co-purified with mutant protein, MALDI-TOF MS analysis of the 11 kDa band excised from the SDS–PAGE was performed. Trypsin digestion and MALDI-TOF MS analysis of the 11 kDa band showed peptides arising from both NH_2_- and COOH-terminal regions of αA-crystallin protein and accounted for 47% of the native αA-crystallin sequence, confirming that the mutant protein was the source of the 11 kDa band. Filtration of the 0.1 mg/ml αAG98R solution through 50 kDa filter separated the oligomers from monomers. This was confirmed by size exclusion chromatography of 50 kDa filtrate using TSK 3000 gel filtration column and using as standards BSA (66 kDa), carbonic anhydrase (29 kDa) and lysozyme (14.6 kDa). As shown in Figure 3, the Ve/Vo value for αAG98R-crystallin collected as filtrate during 50 kDa filtration was 4.29, which corresponds to a molecular weight of 19,500 Da, estimated using the elution profile of standard proteins – serum albumin, carbonic anhydrase and lysozyme.

**Figure 4:**
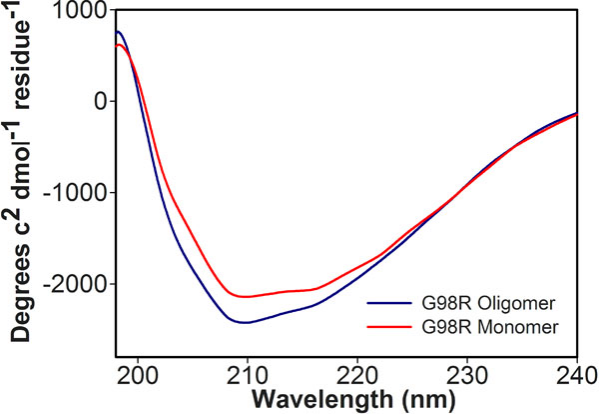
Far-UV Circular dichroism (CD) spectra of αAG98R-crystallin monomeric protein. CD spectra were recorded using 0.1 mg/ml protein in a 0.2 cm cell path length cuvette at 25 °C. The spectra shown represent an average of six scans.

To determine whether structural changes are present in dissociated αAG98R-crystallin subunits as compared to the wild-type protein, the subunits of the mutant protein collected by filtration were subjected to far-UV CD spectroscopy in Jasco J 815 spectropolarimeter. When the protein sample (0.1 mg/ml) was analyzed in a 0.2-cm cuvette, the spectrum of the monomer sample showed increased negative ellipticity at 208 nm, indicating that the mutant protein has increased α-helical content similar to that of oligomeric αAG98R-crystallin (Figure 4). The secondary structural elements of the monomer protein (α-helix 3.8%, β-sheet 41.3%, β-turn 22.1%, and random coil 32.8%) did not significantly differ from the structural elements of the oligomeric form of αAG98R-crystallin analyzed at the same time (α-helix 3.7%, β-sheet 42.4%, β-turn 22.5%, and random coil 32.5%). However, the secondary structural contents of the mutant protein in both monomeric and oligomeric forms slightly differed from that of wild-type αA-crystallin (α-helix 2.1%, β-sheet 41%, β-turn 22.8%, and random coil 34.1%) [9].

### Chaperone-like activity of monomeric protein

**Figure 5:**
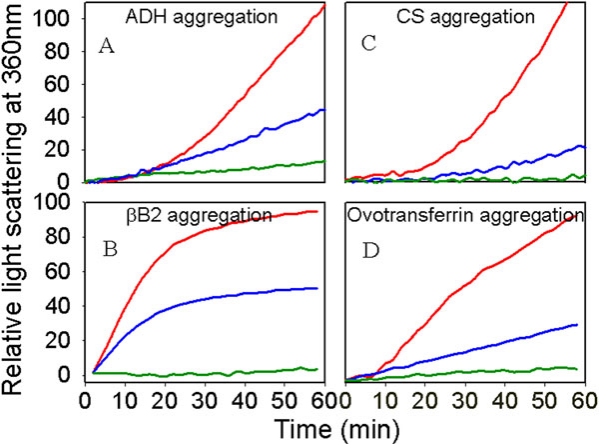
Chaperone activity of αAG98R-crystallin monomers measured with different substrates. The assays were performed as described under methods. Nine μg of the mutant and 10 μg of the wild-type proteins were used in assays. **A**: Thermal aggregation of ADH in the absence or presence of wild-type or αAG98R-crystallin monomer at 37 °C. In each experiment 75 μg of ADH was used. (Red, ADH; blue, + αAG98R-crystallin; green, + wt αA-crystallin). **B**: Thermal aggregation of βB2-crystallin in the presence of αAG98R-crystallin monomers. In each experiment 150 μg of βB2-crystallin was used. (Red, βB_2_; blue, +αAG98R-crystallin; green, + wt αA-crystallin). **C**: Thermal aggregation of CS in the presence and absence of αAG98R-crystallin monomers. In each experiment 75 μg of CS was used. (Red, CS; blue, +αAG98R-crystallin; green,+ wt αA-crystallin). **D**: Thermal aggregation of ovotransferrin in presence or absence of αAG98R-crystallin monomers. In each experiment 100 μg of ovotransferrin was used. (Red, ovotransferrin; blue, +αAG98R-crystallin; green, + wt αA-crystallin).

To investigate whether oligomerization is essential for chaperone activity of αA-crystallin, we performed chaperone assays using monomers of αAG98R-crystallin and the four different client proteins. Aggregation of CS. bovine βB_2_-crystallin, ADH, and ovotransferrin was measured in the presence and absence of αAG98R-crystallin monomers. Monomers of αAG98R-crystallin displayed chaperone activity against EDTA-induced ADH aggregation (Figure 5A). However, the dissociated subunits showed 25% less protection than the oligomeric wild-type protein (Figure 5A). CS aggregation assay showed that the monomers of mutant αAG98R-crystallin have chaperone activity at 43 °C (Figure 5C) and the activity was comparable to that of wild-type oligomer. αAG98R-crystallin monomers also suppressed the aggregation of denaturing ovotransferrin (Figure 5D) and βB2-crsytallin (Figure 5B). However, against denaturing βB2-crystallin and ovotransferrin, the mutant monomers showed 25% and 50%, respectively, lower activity than the wild-type oligomer. In all of the chaperone assays, the activity of αAG98R-crystallin monomers was concentration dependent. Doubling the amount of mutant protein in assays completely suppressed the aggregation of all 4 client proteins.

### Impaired stability of αAG98R-crystallin mutant protein

**Figure 6:**
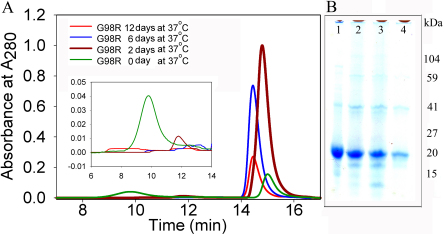
Stability of αAG98R-crystallin. **A**: αAG98R Mutant protein was removed from −80 °C (stored for a year) and size exclusion chromatography was performed at room temperature after 0 – 12 days of incubation of the protein at 0.1 mg/ml at 37 °C. Day 0, Green; day 2, brown; day 6, blue and day 12, red. Inset picture shows enlarged view of the elution profile at the oligomer region, indicating loss of oligomer peak. **B**: SDS–PAGE of samples incubated for 0, 2, 6 and12 days. (Lane 1, 0; lane 2, 2 days; lane 3, 6 days; lane 4, 12 days).

To examine whether stability of the protein in its monomer form is affected by the αAG98R-crystallin mutation, the recombinant G98R protein (100 µg) in 1 ml of 50 mM phosphate buffer containing 150 mM NaCl (pH 7.2) was incubated under sterile conditions in presence of protease inhibitor cocktail at 37 °C for 0, 2, 6, and 12 days. The aggregates formed were removed by centrifugation and the supernatant analyzed by size exclusion chromatography and MALS system. Prior to incubation, αAG98R-crystallin eluted as two peaks: one at the expected oligomer elution region and the other corresponded to the monomer size at 14.5 min (Figure 6A, green line). The non-incubated sample showed an average oligomeric mass of 2,250 kDa. After 2 days of incubation, the oligomeric peak was significantly reduced to a 420 kDa peak (Figure 6A inset). The 280 nm profile showed a decreased oligomeric peak and an increased monomeric peak, indicating that the dissociation of the oligomer into subunits occurred during 2 days of incubation. The samples incubated for 6 days and 12 days showed complete loss of oligomeric peak, probably owing to the precipitation of some of the oligomers, since αAG98R-crystallin is known to precipitate on prolonged incubation [9]. The slight difference among the samples in the elution profile around 14.5 min is likely due to the interaction of breakdown peptides with the monomeric form of the protein. Samples incubated 0 to 12 days were also analyzed by SDS–PAGE without centrifugation to remove the precipitates (Figure 6B). The electrophoretic profile showed a breakdown of αAG98R-crystallin into several <20 kDa size protein bands. There was also some aggregate formation (specifically dimer) in the samples incubated for 2 and 6 days. In the protein sample incubated for 12 days, most of the protein had undergone degradation. The degradation of the protein was confirmed by MALDI TOF MS/MS analysis (data not shown).

## Discussion

A novel αA-crystallin mutation, G98R, is known to cause early-onset cataract [7]. We [9] and others [8] have reported that the αAG98R-crystallin mutant protein forms larger oligomers of a polydisperse nature and exhibits lower thermal stability than the wild-type αA-crystallin. At concentrations below 1 mg/ml, the mutant protein begins to dissociate into a monomeric form. The monomers of αAG98R-crystallin can be separated from the oligomers by size exclusion chromatography (Figure 2A) or by filtration using molecular weight cutoff filters. Surprisingly, the oligomers of intermediate size (between1000 kDa and 20 kDa) were undetectable during gel chromatography (Figure 2A). We found that the dissociation of αAG98R-crystallin oligomer was maximal around pH 5.9. Under similar experimental conditions, the wild-type αA-crystallin did not dissociate into monomers. The findings of our present study confirm earlier observation [2] that wild-type αA- does not dissociate into monomers under physiologic conditions but demonstrate that mutant form does dissociate into monomers at low concentrations. It is known that the truncated αA- and αB-crystallins form oligomers of various sizes [2,4,21,22]. NH_2_-terminally truncated αA^56–173^ and αB^60–175^ crystallins form dimers, trimers, and tetramers [2]. In addition, the αB^66–175^ shows concentration-dependent changes in oligomer size. At 0.07 mg/ml concentration, αB^66–175^ elutes as mostly dimers and trimers, whereas at ~3.3 mg/ml concentration, the protein elutes as high-molecular weight oligomers [2]. The myopathy-causing truncated αB-crystallin, αBQ151X, elutes from a Superose 6 column with characteristics similar to those of a monomeric state [4]. Earlier it was reported that native α-crystallin at low concentrations (0.1 mg/ml) dissociates into monomers [3] but this was contradictory to another study [2] that showed no dissociation of oligomers. However, dissociation of α-crystallin into monomers has also been observed during nanoelectrospray MS studies [23] which could be characterized as non-physiologic conditions. The dissociation of sHSP 27 into <70 kDa oligomers following phosphorylation [24] or into dimers after R148G mutation [25] and to monomers after R127W and S135F mutation [26] has been reported. Previous studies have also suggested that sHSPs dimers are likely the building blocks of oligomers [27,28]. To our knowledge there are no reports of a full-length αA-crystallin mutant that dissociates into monomers under physiologic conditions.

The G98R mutation resides in the protein at a strand region equivalent to the β5-strand in sHSP 16.5 and αA-crystallin [29]. This substitution introduces a positively charged bulky residue at this subunit interaction region likely affecting the subunit interactions that form the stable oligomers. It is unlikely that the relative increase in pI is responsible for the dissociation of mutant protein since it was reported earlier that αAS173K, with similar change in pI did not dissociate into monomers [30]. Mutation of G98 to a noncharged, less bulkier residue Cys was found to not result in a significant alteration in the oligomer size [29]. Our observation of less αAG98R-crystallin dissociation at higher protein concentrations is likely related to a “molecular crowding effect” in which the subunits tend to interact with one another and remain as oligomers. Molecular crowding is known to affect protein–protein interactions and, at high concentrations, proteins tend to aggregate more [31]. Therefore, although αAG98R-crystallin dissociates into monomeric form at low concentration in vitro, it is unlikely that αAG98R-crystallin exists in the lens as a monomer in vivo because of high protein concentration and the propensity to form large aggregates.

α-Crystallin chaperone activity in the lens has been investigated in both wild-type and mutant αA- and αB-crystallins. Chaperone activity is dependent on the rate of subunit exchange, a characteristic of sHSPs [32]. Studies with Hsp27 and T4 lysozyme showed that dissociation of oligomer is required for client protein binding [33]. Dimers have been identified as the preferred chaperone units in sHSPs [11,33,34]. Several reports suggest that subunit dissociation is required for better chaperone activity [26,32-34]. However, in another study, α-crystallin chaperone activity did not change after crosslinking to prevent the dissociation [35]. Our studies with αAG98R-crystallin show that the monomeric form of mutant protein displays chaperone activity toward aggregating of ADH, bovine βB2-crystallin, CS, and ovotransferrin. Among these 4 client proteins, ADH, CS, and bovine βB2-crystallin were denatured by heat, whereas ovotransferrin was denatured by reduction. The results show that the mutant is active against client proteins denatured by heat or chemical methods. The monomers of αAG98R-crystallin showed varying amounts of chaperone activity during aggregation assays with CS, βB2-crystallin, and ovotransferrin as client proteins. While there was nearly a 50% protection of βB2-crystallin with monomers of αAG98R-crystallin when used in 5:1 ratio (w/w), the same ratio between ovotransferrin and αAG98R-crystallin was found to give nearly 100% protection from aggregation (Figure 5B,D). Such a wide range in chaperone activity in both mutant and wild-type αA-crystallin has been observed in other studies [8,9,14,18,21]. The reasons for such substrate-dependent chaperone activity are yet to be elucidated. It is plausible that the mutation affects one of the chaperone binding sites involved in interactions with a particular client protein, whereas the interactions with another client protein and chaperone are not altered because a different binding site is involved. Earlier we found that ^89^VLGDVIEVHGK^99^ [36] is one of the substrate binding sites in αA-crystallin and that the G98R mutation is part of this region. The scientific literature on α-crystallin chaperone activity point to multiple regions of the protein as having a role in chaperone activity. To our knowledge the present study is the first demonstration of chaperone activity in an isolated full-length αA-crystallin monomer.

The monomers of αAG98R-crystallin mutant protein showed lower chaperone activity than wild-type αA-crystallin. A factor may be that the complexes of ADH and αAG98R-crystallin monomers are less stable than the complexes formed with wild-type αA-crystallin and lead to the formation of light scattering aggregates with time. SDS–PAGE analysis of the aggregates supports this view (data not shown). While this study demonstrates chaperone activity in monomers of αAG98R-crystallin, it does not clarify the necessity of subunit dissociation for chaperone activity. Our study does, however, provide enough support for the argument that a subunit in αA-crystallin is a functional unit and dimers are not the active minimum chaperone units in αA-crystallin. Recent studies with mutant forms of HSPB1 (HSP27) found that monomerization of the protein leads to hyperactivity [26]. However, unlike αAG98R-crystallin, HSPB1 mutants only partially dissociated into monomers.

Studies have shown that some mutations affect the size and stability of αA- and αB-cystallin oligomers, but other mutations do not [2,8,9,14,16,22]. The present study reveals that αAG98R-crystallin undergoes autolysis on storage, as has been reported with αBR120G-crystallin [19]. It is possible that trace amount of co-purified protease may cause the degradation of recombinant proteins. However, it is unlikely that the degradation we observed was due to protease contamination because the wild-type αA-crystallin expressed, purified and stored under similar conditions did not show degradation. Degradation of aggregated lipase, γ-crystallin and scFv protein (single-chain variable fragment antibody) has been reported [37], and the autolysis was attributed to the action of surface serine residues in the aggregated proteins. It is yet to be determined whether aggregation-induced proteolysis and serine residues are also involved in αAG98R-crystallin autolysis.

In conclusion, the present study shows that the monomers of αAG98R-crystallin have chaperone activity and that G98R mutation in αA-crystallin is sufficient to cause the dissociation of the oligomers.

## References

[1] Groenen PJ Merck KB de Jong WW Bloemendal H Structure and modifications of the junior chaperone alpha-crystallin. From lens transparency to molecular pathology. Eur J Biochem 1994 225 1 19 7925426 10.1111/j.1432-1033.1994.00001.x

[2] Horwitz J Alpha crystallin: the quest for a homogeneous quaternary structure. Exp Eye Res 2009 88 190 4 18703051 10.1016/j.exer.2008.07.007 PMC2678943

[3] Doss EW Ward KA Koretz JF Preliminary studies on the aggregation process of alpha-crystallin. Exp Eye Res 1997 65 255 66 9268594 10.1006/exer.1997.0337

[4] Hayes VH Devlin G Quinlan RA Truncation of alphaB-crystallin by the myopathy-causing Q151X mutation significantly destabilizes the protein leading to aggregate formation in transfected cells. J Biol Chem 2008 283 10500 12 18230612 10.1074/jbc.M706453200 PMC2447664

[5] Horwitz J Alpha-crystallin can function as a molecular chaperone. Proc Natl Acad Sci USA 1992 89 10449 53 1438232 10.1073/pnas.89.21.10449 PMC50356

[6] Graw J The crystallins: genes, proteins and diseases. Biol Chem 1997 378 1331 48 9426193

[7] Santhiya ST Soker T Klopp N Illig T Prakash MV Selvaraj B Gopinath PM Graw J Identification of a novel, putative cataract-causing allele in CRYAA (G98R) in an Indian family. Mol Vis 2006 12 768 73 16862070

[8] Singh D Raman B Ramakrishna T Rao Ch M The cataract-causing mutation G98R in human alphaA-crystallin leads to folding defects and loss of chaperone activity. Mol Vis 2006 12 1372 9 17149363

[9] Murugesan R Santhoshkumar P Sharma KK Cataract-causing alphaAG98R mutant shows substrate-dependent chaperone activity. Mol Vis 2007 13 2301 9 18199971

[10] Horwitz J Huang Q Ding L The native oligomeric organization of alpha-crystallin, is it necessary for its chaperone function? Exp Eye Res 2004 79 817 21 15642318 10.1016/j.exer.2004.05.007

[11] Feil IK Malfois M Hendle J van Der Zandt H Svergun DI A novel quaternary structure of the dimeric alpha-crystallin domain with chaperone-like activity. J Biol Chem 2001 276 12024 9 11278766 10.1074/jbc.M010856200

[12] Bhattacharyya J Padmanabha Udupa EG Wang J Sharma KK Mini-alphaB-crystallin: a functional element of alphaB-crystallin with chaperone-like activity. Biochemistry 2006 45 3069 76 16503662 10.1021/bi0518141 PMC2615690

[13] Sharma KK Kumar RS Kumar GS Quinn PT Synthesis and characterization of a peptide identified as a functional element in alphaA-crystallin. J Biol Chem 2000 275 3767 71 10660525 10.1074/jbc.275.6.3767

[14] Shroff NP Cherian-Shaw M Bera S Abraham EC Mutation of R116C results in highly oligomerized alpha A-crystallin with modified structure and defective chaperone-like function. Biochemistry 2000 39 1420 6 10684623 10.1021/bi991656b

[15] Bhagyalaxmi SG Srinivas P Barton KA Kumar KR Vidyavathi M Petrash JM Bhanuprakash RG Padma T A novel mutation (F71L) in alphaA-crystallin with defective chaperone-like function associated with age-related cataract. Biochim Biophys Acta 2009 1792 974 81 19595763 10.1016/j.bbadis.2009.06.011 PMC3816373

[16] Pang M Su JT Feng S Tang ZW Gu F Zhang M Ma X Yan YB Effects of congenital cataract mutation R116H on alphaA-crystallin structure, function and stability. Biochim Biophys Acta 2010 1804 948 56 20079887 10.1016/j.bbapap.2010.01.001

[17] Kumar LV Ramakrishna T Rao CM Structural and functional consequences of the mutation of a conserved arginine residue in alphaA and alphaB crystallins. J Biol Chem 1999 274 24137 41 10446186 10.1074/jbc.274.34.24137

[18] Michiel M Skouri-Panet F Duprat E Simon S Ferard C Tardieu A Finet S Abnormal assemblies and subunit exchange of alphaB-crystallin R120 mutants could be associated with destabilization of the dimeric substructure. Biochemistry 2009 48 442 53 19140694 10.1021/bi8014967

[19] Treweek TM Rekas A Lindner RA Walker MJ Aquilina JA Robinson CV Horwitz J Perang MD Quinlan RA Carver JA R120G alphaB-crystallin promotes the unfolding of reduced alpha-lactalbumin and is inherently unstable. FEBS J 2005 272 711 24 15670152 10.1111/j.1742-4658.2004.04507.x

[20] Santhoshkumar P Udupa P Murugesan R Sharma KK Significance of interactions of low molecular weight crystallin fragments in lens aging and cataract formation. J Biol Chem 2008 283 8477 85 18227073 10.1074/jbc.M705876200 PMC2417163

[21] Li Y Schmitz KR Salerno JC Koretz JF The role of the conserved COOH-terminal triad in alphaA-crystallin aggregation and functionality. Mol Vis 2007 13 1758 68 17960114

[22] Rajan S Chandrashekar R Aziz A Abraham EC Role of arginine-163 and the 163REEK166 motif in the oligomerization of truncated alpha A-crystallins. Biochemistry 2006 45 15684 91 17176090 10.1021/bi060705z

[23] Aquilina JA Benesch JL Ding LL Yaron O Horwitz J Robinson CV Subunit exchange of polydisperse proteins: mass spectrometry reveals consequences of alphaA-crystallin truncation. J Biol Chem 2005 280 14485 91 15701626 10.1074/jbc.M500135200

[24] Kato K Hasegawa K Goto S Inaguma Y Dissociation as a result of phosphorylation of an aggregated form of the small stress protein, hsp27. J Biol Chem 1994 269 11274 8 8157658

[25] Chávez Zobel AT Lambert H Theriault JR Landry J Structural instability caused by a mutation at a conserved arginine in the alpha-crystallin domain of Chinese hamster heat shock protein 27. Cell Stress Chaperones 2005 10 157 66 16038412 10.1379/CSC-102.1 PMC1176474

[26] Almeida-Souza L Goethals S deWinter V Dierick I Gallardo R Van Durme J Irobi J Gettemans J Rousseau F Schymkowitz J Timmerman V Janssens S Increased monomerization of mutant HSPB1 leads to protein hyperactivity in Charcot-Marie-Tooth neuropathy. J Biol Chem 2010 285 12778 86 20178975 10.1074/jbc.M109.082644 PMC2857091

[27] Dudich IV Zav’yalov VP Pfeil W Gaestel M Zav’yalova GA Denesyuk AI Korpela T Dimer structure as a minimum cooperative subunit of small heat-shock proteins. Biochim Biophys Acta 1995 1253 163 8 8519797 10.1016/0167-4838(95)00135-x

[28] Bagnéris C Bateman OA Naylor CE Cronin N Boelens WC Keep NH Slingsby C Crystal structures of alpha-crystallin domain dimers of alphaB-crystallin and Hsp20. J Mol Biol 2009 392 1242 52 19646995 10.1016/j.jmb.2009.07.069

[29] Koteiche HA Berengian AR McHaourab HS Identification of protein folding patterns using site-directed spin labeling. Structural characterization of a beta-sheet and putative substrate binding regions in the conserved domain of alpha A-crystallin. Biochemistry 1998 37 12681 8 9737844 10.1021/bi9814078

[30] Derham BK van Boekel MAM Muchowski PJ Clark JI Horwitz J Hepburne-Scott HW de Jong ww, Crabbe MJC, Harding JJ. Chaperone function of mutant versions of αA- and αB-crystallin prepared to pinpoint chaperone binding sites. Eur J Biochem 2001 268 713 21 11168410 10.1046/j.1432-1327.2001.01929.x

[31] Minton AP The influence of macromolecular crowding for protein assembly. Curr Opin Struct Biol 2000 10 34 9 10679465 10.1016/s0959-440x(99)00045-7

[32] Stengel F Baldwin AJ Painter AJ Jaya N Basha E Kay LE Vierling E Robinson CV Benesch JL Quaternary dynamics and plasticity underlie small heat shock protein chaperone function. Proc Natl Acad Sci USA 2010 107 2007 12 20133845 10.1073/pnas.0910126107 PMC2836621

[33] Shashidharamurthy R Koteiche HA Dong J McHaourab HS Mechanism of chaperone function in small heat shock proteins: dissociation of the HSP27 oligomer is required for recognition and binding of destabilized T4 lysozyme. J Biol Chem 2005 280 5281 9 15542604 10.1074/jbc.M407236200

[34] Giese KC Vierling E Changes in oligomerization are essential for the chaperone activity of a small heat shock protein in vivo and in vitro. J Biol Chem 2002 277 46310 8 12297515 10.1074/jbc.M208926200

[35] Augusteyn RC Dissociation is not required for alpha-crystallin's chaperone function. Exp Eye Res 2004 79 781 4 15642315 10.1016/j.exer.2004.08.010

[36] Sharma KK Kumar GS Murphy AS Kester K Identification of 1,1'-bi(4-anilino)naphthalene-5,5′-disulfonic acid binding sequences in alpha-crystallin. J Biol Chem 1998 273 15474 8 9624133 10.1074/jbc.273.25.15474

[37] Sharma M Luthra-Guptasarma M. Degradation of proteins upon storage at near-neutral pH: indications of a proteolytic/gelatinolytic activity associated with aggregates. Biochim Biophys Acta 2009 1790 1282 94 19563865 10.1016/j.bbagen.2009.06.010

